# Community-Based Activity and Sedentary Patterns Are Associated With Cognitive Performance in Mobility-Limited Older Adults

**DOI:** 10.3389/fnagi.2018.00341

**Published:** 2018-11-15

**Authors:** Amal A. Wanigatunga, Todd M. Manini, Delilah R. Cook, Jeffrey Katula, Roger A. Fielding, Arthur F. Kramer, Joe Verghese, Stephen R. Rapp, Kaycee M. Sink, Abby C. King, Thomas W. Buford, Steve Anton, Neelesh Nadkarni, Janine M. Jennings, Kieran Reid, Mark A. Espeland, Thomas M. Gill, Marco Pahor, Joe R. Nocera

**Affiliations:** ^1^Johns Hopkins School of Public Health, Baltimore, MD, United States; ^2^Department of Aging and Geriatric Research, University of Florida, Gainesville, FL, United States; ^3^Division of Public Health Sciences, Department of Health and Exercise Science, Wake Forest University, Winston-Salem, NC, United States; ^4^Department of Health and Exercise Science, Wake Forest University, Winston-Salem, NC, United States; ^5^Jean Mayer USDA Human Nutrition Research Center on Aging, Tufts University, Boston, MA, United States; ^6^Department of Psychology, University of Illinois at Urbana–Champaign, Champaign, IL, United States; ^7^Department of Neurology and Medicine, Albert Einstein of Yeshiva University, Bronx, NY, United States; ^8^Department of Psychiatry and Behavioral Medicine, Wake Forest School of Medicine, Winston-Salem, NC, United States; ^9^Department of Internal Medicine (Gerontology/Geriatrics), Wake Forest School of Medicine, Winston-Salem, NC, United States; ^10^Stanford University School of Medicine, Stanford, CA, United States; ^11^School of Medicine, The University of Alabama at Birmingham, Birmingham, AL, United States; ^12^Department of Medicine, University of Pittsburgh, Pittsburgh, PA, United States; ^13^Department of Psychology, Wake Forest University, Winston-Salem, NC, United States; ^14^Department of Biostatistical Sciences, Wake Forest School of Medicine, Winston-Salem, NC, United States; ^15^Yale School of Medicine, New Haven, CT, United States; ^16^Center for Visual and Neurocognitive Rehabilitation, Atlanta, GA, United States; ^17^Division of Physical Therapy, Emory University, Atlanta, GA, United States; ^18^Department of Neurology, Emory University, Atlanta, GA, United States

**Keywords:** accelerometer, wearables, executive function, cognition, aging, physical inactivity

## Abstract

Over the last few decades, considerable evidence shows that greater levels of aerobic exercise and cardiovascular fitness benefit cognitive performance. However, the degree to which free-living activity in community settings is related to cognitive performance remains unclear, particularly in older adults vulnerable to disability. Also, it is unknown whether the manner in which daily physical activity (PA) and sedentary time are accumulated throughout the day is associated with cognition. Cross-sectional associations between accelerometer-characterized PA and sedentary patterns and cognitive performance were examined in 1,274 mobility-limited older adults. Percent time spent in various bout lengths of PA (≥1, ≥2, and ≥5 min) and sedentary (≥1, ≥30, and ≥60 min) was defined as the number of minutes registered divided by total wear time × 100. Percent time was then tertiled for each bout length. Multiple linear regression models were used to estimate the associations between accelerometer bout variables and separate cognitive domains that included processing speed (Digit Symbol Coding; DSC), immediate and delayed recall (Hopkins Verbal Learning Test; HVLT), information processing and selective attention (Flanker), working memory (*n*-back), reaction time (switch and non-switch reaction time), and a composite score that averaged results from all cognitive tests. After adjusting for demographics, behavioral factors, and morbid conditions, more time spent in PA was associated with higher DSC for all bout lengths (*p* < 0.03 for all). Higher PA was associated with higher HVLT and global cognition scores but only for longer bout lengths (*p* < 0.05 for all). The association was largely driven by participants who spent the lowest amount of time performing activity while awake (*p* < 0.04). An inverse linear relationship was observed between total sedentary time and DSC (*p* = 0.02), but not for other measures of cognition. These results suggest that, while higher PA was associated with higher cognitive performance, PA’s association with memory was sensitive to bout duration. The time, but not the manner, spent in sedentary behaviors showed a minor association with executive function. Further research is warranted to characterize longitudinal changes in daily activity and sedentary patterns as potential biophysical markers of cognitive status in older adults.

## Introduction

Due to the growing aging population within the United States, there is considerable interest in identifying lifestyle interventions that can enhance brain health and potentially reverse and/or prevent cognitive decline. Over the last few decades, both cross-sectional and longitudinal evidence suggest that higher physical activity (PA) is associated with higher cognitive performance (Kramer et al., 1999; Voss et al., 2013; Nocera et al., 2015), higher brain volume (Colcombe et al., 2003; Erickson et al., 2011), and higher brain efficiency as measured by functional magnetic resonance imaging (MRI) (McGregor et al., 2012, 2013). Additionally, activity-based interventions appear to have positive effects on outcomes including memory, processing speed, and executive function (Kramer et al., 1999; Colcombe and Kramer, 2003; Voss et al., 2013; Nocera et al., 2015). While PA remain a major factor in promoting brain health, there is increasing interest in understanding the unique role of sedentary behavior.

Chronic and excessive time spent being sedentary are associated with deleterious effects on cardiovascular and metabolic outcomes, regardless of PA engagement (Biswas et al., 2015). Vascular and metabolic dysfunction has been shown to result in amyloid-β buildup (Vemuri et al., 2015), blood brain barrier deterioration (Zhao et al., 2015), arterial stiffness (Hughes et al., 2018), endothelial dysfunction (Guerra et al., 2018), and cerebrovascular abnormalities (Yew et al., 2017), and are likely major pathways leading to cognitive decline (Kennedy et al., 2016; Strickland, 2018). Although participating in structured PA routinely can mitigate these negative effects, older adults have low exercise participation (Keadle et al., 2016), especially among older adults living with mobility disability (Manns et al., 2015). Further, older adults with mobility disability not only have lower overall activity and more sedentary, but also perform activity in shorter durations while engaging in long, uninterrupted bouts of sedentary time throughout the day (Manns et al., 2015). Therefore, targeting the manner of which PA and sedentary time is performed may be a complementary approach to increasing overall PA to improve cardiometabolic health and maintain cognitive abilities in an effort to prevent disability.

Portable activity monitors such as accelerometers objectively capture both time spent in PA and sedentary behaviors continuously throughout the day. Not only can accelerometer data be utilized to quantify daily volumes (e.g., total accumulated amount of sedentary time), they can be also used to characterize accrual patterns into bouts (Wanigatunga et al., 2017a,b). Emerging evidence shows that accelerometer-derived PA tends to be associated with higher cognitive performance across executive function, memory, attention, and fluency (Menai et al., 2017; Zhu et al., 2017; Iso-Markku et al., 2018) while accelerometer-derived sedentary time is linked with executive function (Vásquez et al., 2017). However, little work has been done to characterize patterns in which PA and sedentary times are accrued throughout the day in relation to cognition. This is important because current PA guidelines promote the engagement of multiple activity intervals or bouts to reach 30 min/day of moderate-to-vigorous PA for older adults, particularly those vulnerable to disability (Nelson et al., 2007). Further, older adults are the most sedentary of US adults (Evenson et al., 2012), and this may predispose them to a higher rate of adverse health outcomes, especially when engaging in prolonged sedentary behavior (Healy et al., 2008, 2011). Coupled with the rising healthcare burden due to dementia and associated cognitive decline (Wimo et al., 2017), understanding the relationship between objectively measured PA and sedentary times (and the patterns in which these lifestyle behaviors are accrued) and domain-specific and global cognitive performance is critical to the maintenance of health and well-being in older adults.

The purpose of this study was to evaluate the cross-sectional association between accrual patterns of PA and sedentary time with cognitive performance in mobility-limited older adults. The first aim was to assess whether total PA and accrual patterns in short and long bout lengths of PA were associated with multiple domains of cognitive performance. We hypothesized that total PA, particularly PA time spent in longer bout durations, were associated with higher cognitive performance in mobility-limited older adults. The second aim examined the association of total sedentary time and accrual of sedentary time in various bout lengths with multiple domains of cognitive performance. We hypothesized that higher amounts of sedentary time, particularly in prolonged bouts, were associated with lower cognitive performance in mobility-limited older adults.

## Materials and Methods

### Study Design and Sample

Baseline accelerometer and cognitive data were utilized from the Lifestyle Interventions and Independence for Elders (LIFE) Study. Details about specific study inclusion and exclusion criteria of the LIFE study have been reported previously (Fielding et al., 2011). Briefly, participants were eligible if they were 70–89 years of age, were at a high risk for mobility disability based on a summary of performance tests meant for older adults (Guralnik et al., 1994), and reported being sedentary defined as spending less than 20 min per week in moderate-to-vigorous PA as assessed by the Community Healthy Activities Model Program for Seniors (CHAMPS) PA questionnaire (Rejeski et al., 2013). Participants needed to have sufficient cognitive abilities to both perform assessments and to provide informed consent to the study. Therefore, those with 9 or more years of education who score <80 (<76 if African American) out of 100 on the Modified Mini-Mental Status Examination (3MSE) (Teng and Chui, 1987) and those with less than 9 years education who score <76 (<70 if African American or Spanish speaking) were excluded. A total of 1,635 older adults were recruited at eight US field centers (Pahor et al., 2014; Sink et al., 2014). Several committees (steering, recruitment and retention, outcome and assessment, and field operations) were responsible for establishing and monitoring ethics of the trial. Additionally, a data and safety monitoring board were responsible for overseeing the LIFE study across all eight participating institutions. Each institution obtained human subjects committee approval and all participants provided informed consent in accordance with the Declaration of Helsinki that was confirmed by the coordinating center. The study protocol is available on request at www.thelifestudy.org/public/index.cfm. The trial is registered with ClinicalTrials.gov with the identifier NCT01072500.

### Accelerometry

The hip-worn, Actigraph tri-axial accelerometer (Model GT3X; ActiGraph^TM^) was used to objectively measure time spent in PA and sedentary levels. Participants were instructed to wear the accelerometer for a minimum of 7 consecutive days immediately following their baseline clinic visit. Additionally, participants were instructed to wear the device when they were awake and to remove the device during sleep and water-based activities such as showering. Movements were recorded as activity counts (unit-less quantities of movement) collected in 1-s epochs and then later binned into counts per minute. Non-wear time was defined as a 90-min window of zero counts in the vertical axis, allowing for up to 2-min of non-zero counts <100 counts/min and removed for the analysis (Choi et al., 2011). A valid accelerometer collection period per participant was defined as wearing the accelerometer for ≥10 h/day (valid day) for ≥3 days. Out of 1,635 men and women recruited for the LIFE study, 1,341 participants had valid accelerometer data.

Each minute in the accelerometer data was labeled as either a sedentary (<100 counts/min) or an activity (≥100 counts/min) minute. Bouts were defined as consecutive minutes registering in a specific activity level (i.e., sedentary or active). Three bout lengths were used for activity (≥1, ≥2, and ≥5 min bout lengths) and for sedentary (≥1, ≥30, and ≥60 min bout lengths) time. Since more time registered as sedentary than active, longer sedentary bout lengths were used as previously reported (Wanigatunga et al., 2017a,b). The percentage of time spent in each bout length was calculated as follows: (average minutes/day spent within a bout length)/(average minutes/day of wear time) × 100. In statistical models, percent time was treated continuously and categorically where the percentage of time spent at each bout length was tertiled to compare low, medium, and high amounts of activity or sedentary time.

### Cognitive Performance

The cognitive test battery consisted of a test of psychomotor speed, attention, and working memory [Wechsler Adult Intelligence Scale-III Digit Symbol Coding (DSC) (Wechsler, 1997)] and 12-item word list learning and recall tasks [Hopkins Verbal Learning Test (HVLT)-Revised (Brandt and Benedict, 2001)]. For added sensitivity in assessing speed of processing and executive function, a computerized *n*-back (1-back and 2-back), a task switching paradigm (reaction times with switching and no-switching), and the Erickson flanker task (reaction times under congruent and incongruent conditions) were also administered (Sink et al., 2014). These measures were chosen as each tap into an aspect of executive function that has previously been shown to improve in older adults following a PA intervention designed to enhance aerobic fitness and functional capacity (Kramer et al., 2001; Colcombe and Kramer, 2003). A practice session was held for the task-based assessments to allow the participant to get comfortable and understand the directions. For the *n*-back task, participants were required to use working memory to identify items presented *n*th places (i.e., 1 or 2) back with percent correct identifications on each utilized in the analyses. For example, the “1-back” test consisted of a one-block trial of 46 letters, 45 of which the participant responded to (no response for the first letter that appears) while the “2-back” test consists of a one-block trial of 47 letters/trials, 45 of which the participant responded to (no response for the first two letters). The flanker task measured selective attention and the ability to inhibit distracting information. Each participant was shown a central arrow and was instructed to indicate which direction it was pointing. During the flanker task, the central arrows are flanked by either congruent (arrows pointing in the same direction) or incongruent items (arrows pointing in the opposite direction). Flanker test consists of one-block with 80 sets of arrows (e.g., 80 trials), with equal numbers of congruent and incongruent items. Reaction times for correct responses were measured for both congruent and incongruent conditions and included as outcomes of interest for analyses. The task switching assessment measured attentional flexibility by having participants alternate between performing two different tasks using the same stimuli (letter/digit pairs). Participants were asked to make a judgment about either the letter or the digit, and consecutive trials required the same (nonswitch trials) or the other judgment task (switch trials); there were equal numbers of nonswitch and switch trials. Similar to the flanker task, reaction times were calculated for correct responses on switch and nonswitch trials and included as outcomes of interest. The task switching measure consisted of 120 trials within one block. Lastly, a standardized global composite score was created as previously described (Sink et al., 2015). Briefly, *z*-scores were calculated for each cognitive test score by dividing the difference between individual and mean score by the standard deviation. A pooled score was formed by averaging the standardized scores of all nine cognitive-test outcomes [across the DSC, HVLT immediate, and delayed; 1- and 2-back; task switching (switch and no-switch conditions); and flanker (congruent and non-congruent conditions) tests]. The pooled score was re-standardized and used for statistical modeling.

### Covariates

Participants self-reported age, sex, race/ethnicity, education, income, and marital status. Body mass index (kg/m^2^) was calculated using height (m) on a stadiometer and weight (kg) on a balance-beam scale. Other covariates include self-described smoking status, sleep quality using the Pittsburgh Sleep Quality Index (Buysse et al., 1991) where lower scores indicate better sleep quality, and overall stress levels using the Perceived Stress Scale (Cohen et al., 1983) where higher scores indicate higher perceived stress; factors known to be associated with both daily activity (Kredlow et al., 2015; Awick et al., 2017; Swan et al., 2018) and cognitive performance (Ott et al., 2004; Aggarwal et al., 2014; Yaffe et al., 2014). Further, a comorbidity index variable was created by summing self-reported history of myocardial infarction, congestive heart failure, stroke, hypertension, cancer, diabetes, arthritis, and lung disease.

### Statistical Approach

Differences in participant characteristics, cognitive scores, and accelerometer wear metrics were tested by either *t*-tests (continuous variables) or chi-squared tests (categorical variables) across tertiles of percent PA time. Means and SDs for daily time (minutes/day) and percent (%) spent in ≥1 (total), ≥2, and ≥5 active minute bouts as well as ≥1 (total), ≥30, and ≥60 sedentary minute bouts were described. Multiple linear regression models were constructed to estimate the association between each accelerometer bout length (treated as a categorical variable of tertiles of daily percentage) and each cognitive variable. Additionally, percent variables were treated as continuous to test for linear trends. Covariates included were age, sex, race/ethnicity, education, income, marital status, BMI, smoking status, sleep quality, perceived stress, and living with two or more morbid conditions.

## Results

Among 1,341 participants who had valid accelerometer data, 39 (3%) did not have any cognitive data and were excluded (final sample *n* = 1,275). On average, participants were 79 (*SD* = 5) years old, 67% were women, 76% were non-Hispanic white, and were overweight with a BMI around 30 kg/m^2^ (Table 1). Across tertiles of higher PA, participants tended to be younger (*p* < 0.001), men (*p* < 0.001), and report lower education (*p* = 0.009), lower income (*p* = 0.003), and lower comorbidities (*p* < 0.001). Additionally, there were higher scores for DSC (*p* < 0.001), higher number of words recalled for HVLT immediate (*p* = 0.002) and delayed (*p* = 0.002) tests, and higher global cognitive scores (*p* = 0.016) across tertiles of higher PA. Although the number of valid wear days was similar between tertiles, average wear time throughout the day was slightly lower across higher PA tertiles (*p* = 0.016).

**Table 1:** Participant characteristics stratified by physical activity (PA)-based tertiles, *n* = 1,275.

	Low (*n* = 425)	Medium (*n* = 425)	High (*n* = 425)	*p*-value
Age (years), mean (*SD*)	79.9 (5.2)	78.9 (5.4)	77.5 (5.0)	<0.001
Female, *n* (%)	225 (52.9)	294 (69.2)	332 (78.1)	<0.001
Non-Hispanic white, *n* (%)	340 (80.0)	329 (77.4)	303 (71.3)	0.225
Beyond HS education, *n* (%)	297 (69.9)	265 (62.4)	248 (58.4)	0.009
$35 k or less annual income, *n* (%)	139 (32.7)	168 (39.5)	187 (44.0)	0.003
Married, *n* (%)	181 (42.6)	150 (35.3)	145 (34.1)	0.071
Body mass index (kg/m^2^), mean (*SD*)	30.1 (6.3)	30.7 (6.0)	30.0 (5.8)	0.823
Current smoker, *n* (%)	15 (3.5)	15 (3.5)	10 (2.4)	0.611
Pittsburgh Sleep Quality Index, mean (*SD*)	5.7 (3.8)	6.0 (3.8)	6.0 (3.9)	0.158
Perceived Stress Scale, mean (*SD*)	10.6 (6.1)	11.2 (6.3)	11.2 (5.7)	0.108
Two or more comorbidities, *n* (%)	127 (29.9)	130 (30.6)	75 (17.7)	<0.001
One-back, % correct, mean (*SD*)	0.81 (0.18)	0.81 (0.18)	0.83 (0.16)	0.125
Two-back, % correct, mean (*SD*)	0.50 (0.21)	0.52 (0.20)	0.51 (0.21)	0.755
Digit Symbol Coding, # correct, mean (*SD*)	43.9 (12.3)	46.9 (12.5)	47.7 (13.3)	<0.001
Task switching–No switch reaction time (ms), mean (*SD*)	1,519.1 (1,075.1)	1,450.0 (1,060.6)	1,447.3 (803.4)	0.321
Task switching–Switch reaction time (ms), mean (*SD*)	2,473.2 (1,323.2)	2,426.8 (1,287.8)	2,385.2 (1,124.2)	0.337
Flanker–Congruent (ms), mean (*SD*)	662.5 (244.8)	656.9 (208.6)	649.9 (209.5)	0.417
Flanker–Incongruent (ms), mean (*SD*)	736.9 (348.5)	737.7 (299.1)	733.7 (303.3)	0.886
HVLT-Immediate recall, # correct, mean (*SD*)	22.7 (5.3)	23.7 (5.3)	23.8 (5.3)	0.002
HVLT-Delayed recall, # correct, mean (*SD*)	7.5 (2.7)	7.8 (2.9)	8.1 (2.8)	0.002
*z*-Composite score, mean (*SD*)	-0.10 (0.70)	-0.01 (0.70)	0.01 (0.70)	0.016
Wear days, mean (*SD*)	8.1 (3.3)	8.3 (3.3)	7.8 (3.0)	0.295
Wear minutes/day, mean (*SD*)	845.5 (125.9)	841.7 (112.6)	827.1 (94.1)	0.016

*HS, high school; HVLT, Hopkins Verbal Learning Test*.

Average time spent in activity and sedentary bout lengths described as either a daily percentage or absolute time (minutes/day) are found in Table 2. On average, LIFE participants in the low, medium, and high tertiles spent 14 (120 min/day), 22 (188 min/day), and 32% (263 min/day) of the day in total daily PA, respectively. Further, those in the low, medium, and high tertiles spent 68 (564 min/day), 78 (654 min/day), and 86% (725 min/day) of the day in total daily sedentary time. Percent time spent in either PA or sedentary time tended to be lower for longer bout lengths.

**Table 2:** Descriptive means and ranges of daily accelerometer metrics by bout-specific tertiles of either total or sedentary activity.

	Low (*n* = 425)		Medium (*n* = 425)		High (*n* = 425)	
	Mean	Min–Max	Mean	Min–Max	Mean	Min–Max
**Total activity volume (≥1-min bouts)**						
%/day	14.3	3.9–18.8	22.3	18.8–25.8	31.9	25.8–60.6
Minutes/day	120.7	29.3–249.1	188.0	123.0–306.2	263.2	167.8–511.8
**≥2-min activity bouts**						
%/day	9.2	1.4–12.7	15.4	12.8–17.9	22.7	17.9–43.9
Minutes/day	77.5	10.3–167.3	129.3	85.7–217.6	187.4	125.5–384.3
**≥5-min activity bouts**						
%/day	2.2	0.2–3.6	4.8	3.7–6.1	8.9	6.1–23.0
Minutes/day	19.0	1.3–42.6	40.8	25.3–77.6	73.2	42.2–206.0
**Total sedentary volume (≥1-min bouts)**						
%/day	68.1	39.3–74.2	77.7	74.2–81.2	85.7	81.2–96.1
Minutes/day	563.8	313.0–938.0	653.6	496.2–1,127.4	724.7	532.0–1,303.9
**≥ 30-min sedentary bouts**						
%/day	19.8	2.9–27.9	34.1	27.9–40.6	51.0	40.7–82.6
Minutes/day	162.7	21.0–313.1	286.4	196.0–536.7	436.5	272.4–922.5
**≥ 60-min sedentary bouts**						
%/day	6.0	0–10.3	15.0	10.3–20.2	29.8	20.2–68.4
Minutes/day	49.5	0–100.2	126.0	69.6–260.2	257.6	144.3–682.6

In fully adjusted models, participants who spent a medium percentage of their time in activity (Table 3, row B) and high percentage of their time in activity (Table 3, row C) in ≥1 activity minute bouts had approximately 2 units higher DSC score when compared to those who spent a low percentage of their activity in ≥1 activity minute bout lengths, respectively (Table 3, row A; *p* < 0.012 for both tertiles). This association with DSC remained relatively stable across ≥2 (Table 3, rows E–G) and ≥5 (Table 3, rows I–K) activity minute bout lengths (*p* < 0.031 for both tertiles and bout lengths).

**Table 3:** Adjusted associations of tertiles of percent activity time by bout length with cognition outcomes, beta-coefficient (SE).

	*n*-back		DSC	Task switching		Flanker		HVLT		Global composite
			
% activity time	One-back (*n* = 1,156)	Two-back (*n* = 1,074)	(*n* = 1,256)	No (*n* = 1,110)	Yes (*n* = 1,110)	Congruent (*n* = 1,213)	Incongruent (*n* = 1,213)	Immediate (*n* = 1,257)	Delayed (*n* = 1,251)	(*n* = 1,258)
**Daily total activity volume (≥1-min bouts)**		
A. Low	Ref.	Ref.	Ref.	Ref.	Ref.	Ref.	Ref.	Ref.	Ref.	Ref.
B. Medium	<0.001 (0.011)	0.012 (0.015)	2.104 (0.827)^∗^	-63.201 (72.822)	-62.972 (91.951)	-16.206 (15.502)	-13.372 (22.447)	0.621 (0.351)	0.113 (0.191)	0.083 (0.046)
C. High	0.011 (0.013)	-0.001 (0.016)	2.030 (0.854)^∗^	-86.220 (74.541)	-117.952 (94.122)	-24.541 (15.993)	-18.602 (23.158)	0.385 (0.363)	0.252 (0.197)	0.085 (0.047)
**Daily ≥2-min activity bouts**		
E. Low	Ref.	Ref.	Ref.	Ref.	Ref.	Ref.	Ref.	Ref.	Ref.	Ref.
F. Medium	0.005 (0.013)	0.018 (0.016)	2.514 (0.826)^∗∗^	-42.912 (72.537)	-49.930 (91.572)	-14.145 (15.482)	-11.805 (22.420)	1.001 (0.350)^∗∗^	0.213 (0.191)	0.105 (0.045)^∗^
G. High	0.016 (0.013)	0.005 (0.016)	1.849 (0.849)^∗^	-99.048 (74.116)	-141.906 (93.566)	-27.796 (15.895)	-23.111 (23.019)	0.476 (0.360)	0.236 (0.196)	0.100 (0.047)^∗^
**Daily ≥5-min activity bouts**		
I. Low	Ref.	Ref.	Ref.	Ref.	Ref.	Ref.	Ref.	Ref.	Ref.	Ref.
J. Medium	-0.006 (0.013)	0.003 (0.015)	2.286 (0.825)^∗∗^	-77.574 (72.30)	-50.018 (91.341)	-24.840 (15.399)	-41.153 (22.278)	1.045 (0.350)^∗∗^	0.386 (0.190)^∗^	0.115 (0.045)^∗^
K. High	0.019 (0.013)	0.015 (0.016)	2.008 (0.845)^∗^	-107.309 (73.880)	-112.888 (93.326)	-23.873 (15.850)	-28.538 (22.931)	0.696 (0.358)	0.387 (0.194)^∗^	0.114 (0.046)^∗^

*DSC, digit symbol coding; HVLT, Hopkins Verbal Learning Test; Ref, reference. Models adjusted for age, sex, race/ethnicity, education, income, marital status, body mass index (kg/m^2^), smoking status, sleep quality, perceived stress, living with two or more morbid conditions. ^∗^*p* < 0.05; ^∗∗^*p* < 0.01; ^∗∗∗^*p* < 0.001*.

For HLVT-immediate, those who spent a medium percentage of their time in activity had an estimated 1 unit score higher in HLVT-immediate when compared to those who spent a low percentage of their activity for ≥2 (*p* < 0.005; Table 3, row F) and ≥5 (*p* < 0.004; Table 3, row J), but not in ≥1 (*p* = 0.077; Table 3, row B) bout lengths.

For HLVT delayed, those who spent a medium percentage of their time in activity (Table 3, row J) and high percentage of their time in activity (Table 3, row K) in ≥5 activity minute bouts reported an estimated 1 unit score higher in HLVT delayed when compared to those with a low percentage, respectively (Table 3, row I; *p* < 0.048 for both tertiles).

Those who spent a medium percentage of their time in activity and high percentage of their time in activity had approximately 0.1 higher standardized global cognitive scores when compared to those with a low percentage of activity, respectively, for both ≥2 (*p* < 0.034 for both tertiles; Table 3, rows F and G) and ≥5 (*p* < 0.015 for both tertiles; Table 3, rows J and K) activity minute bout lengths.

Table 4 shows that only DSC was significantly associated with patterns of sedentary time (Table 4). In fully adjusted models, those who spent a high percentage in sedentary time in ≥1 sedentary minute bouts (Table 4, row C) had 2 (SE = 0.8, *p* = 0.018) units lower DSC score when compared with those who spent a low percentage of sedentary time in ≥1 sedentary minute bouts (Table 4, row A).

**Table 4:** Adjusted associations of tertiles of percent sedentary time by bout length with cognition outcomes, beta-coefficient (SE).

	*n*-back		DSC	Task switching		Flanker		HVLT		Global composite
			
% Sedentary time	One-back (*n* = 1,156)	Two-back (*n* = 1,049)	(*n* = 1,256)	No (*n* = 1,110)	Yes (*n* = 1,110)	Congruent (*n* = 1,213)	Incongruent (*n* = 1,213)	Immediate (*n* = 1,257)	Delayed (*n* = 1,251)	(*n* = 1,258)
**Daily total sedentary volume (≥1-min bouts)**		
A. Low	Ref.	Ref.	Ref.	Ref.	Ref.	Ref.	Ref.	Ref.	Ref.	Ref.
B. Medium	-0.011 (0.013)	0.013 (0.015)	0.073 (0.826)	23.018 (72.112)	54.980 (91.055)	8.336 (15.509)	5.230 (22.457)	0.236 (0.351)	-0.139 (0.190)	-0.002 (0.046)
C. High	-0.012 (0.013)	0.001 (0.016)	-2.030 (0.854)^∗^	86.220 (74.541)	117.953 (94.122)	24.541 (15.994)	18.602 (23.158)	-0.385 (0.363)	-0.252 (0.197)	-0.085 (0.047)
**Daily ≥30-min sedentary bouts**		
E. Low	Ref.	Ref.	Ref.	Ref.	Ref.	Ref.	Ref.	Ref.	Ref.	Ref.
F. Medium	0.006 (0.013)	0.007 (0.016)	-0.170 (0.829)	9.018 (72.465)	-8.939 (91.520)	10.580 (15.497)	3.495 (22.429)	0.355 (0.351)	0.153 (0.190)	0.038 (0.046)
G. High	-0.014 (0.013)	-0.003 (0.016)	-0.519 (0.879)	50.636 (76.635)	27.604 (96.787)	12.798 (16.416)	3.798 (23.759)	0.284 (0.372)	0.199 (0.202)	-0.012 (0.048)
**Daily ≥60-min sedentary bouts**		
I. Low	Ref.	Ref.	Ref.	Ref.	Ref.	Ref.	Ref.	Ref.	Ref.	Ref.
J. Medium	0.001 (0.013)	0.002 (0.015)	-0.539 (0.829)	74.263 (72.442)	13.485 (91.518)	21.624 (15.501)	26.424 (22.435)	0.083 (0.351)	0.150 (0.190)	-0.017 (0.457)
K. High	-0.011 (0.013)	-0.002 (0.016)	-0.330 (0.862)	44.905 (75.231)	29.123 (95.040)	4.761 (16.089)	6.430 (23.285)	0.418 (0.365)	0.113 (0.198)	0.0003 (0.0475)

*DSC, digit symbol coding; HVLT, Hopkins Verbal Learning Test; Ref., Reference. Models adjusted for age, sex, race/ethnicity, education, income, marital status, body mass index (kg/m^2^), smoking status, sleep quality, perceived stress, living with two or more morbid conditions. ^∗^*p* < 0.05; ^∗∗^*p* < 0.01; ^∗∗∗^*p* < 0.001*.

## Discussion

This study demonstrated that higher time spent in PA is associated with higher cognitive performance related to psychomotor speed, attention, and working memory among mobility-limited older adults. Further, greater time spent in longer bout lengths of PA was positively correlated with cognitive domains of working memory and learning as well as overall cognitive performance; a relationship not observed with overall total PA. Interestingly, total sedentary time, but not prolonged sedentary bouts, was found to be negatively associated with cognitive performance related to executive function; a relationship driven by participants with the highest sedentary time. Our findings suggest that, beyond a total summary measure, the manner in which daily PA and sedentary behaviors are accrued may be an important indicator of certain cognitive domains in sedentary older adults.

Evidence that a positive relationship between daily PA and cognition is emerging, particularly for higher intensity activity (Zhu et al., 2017). Two studies support our findings that show positive associations between daily PA and global cognition (Menai et al., 2017; Iso-Markku et al., 2018). In 2017, Menai and colleagues showed that PA collected in short bouts lasting less than 5 min, but not longer bouts, was associated with higher global cognitive function in older adults aged 60–83 years old. We found a similar association between short activity bouts and global cognition, but also found longer activity bouts to be positively associated with global cognition. While there is a large body of evidence that shows acute bouts of exercise (planned PA for health and recreation) have benefits on cognition (Hillman et al., 2008; Barella et al., 2010; Johnson et al., 2016), our results suggest that spending time in PA above sedentary levels, which largely includes purposeful and goal-directed activities (e.g., performing daily chores, walking to get the mail), is associated with higher cognitive performance for mobility-limited older adults (Manini et al., 2006; Middleton et al., 2011). While our findings did not determine PA intensity levels, LIFE participants have been observed to spend most of their activity at very low intensity levels (Wanigatunga et al., 2017b); an intensity level that most older adults reach during activity, primarily due to age-related energy deficits that affect the maintenance of leading an independent lifestyle (Schrack et al., 2010). Further, older adults who spent more time in PA bouts lasting 5 min or longer had higher psychomotor speed, attention, working memory, learning and recall, and global cognition – a finding unique to the literature. Possible biological mechanisms mediating the link between longer PA durations and higher cognition performance include cardiorespiratory maintenance (Kramer and Colcombe, 2018), normal glycemic control (Wheeler et al., 2017), production of brain-derived neurotrophic factor (Håkansson et al., 2017), and reductions in pro-inflammatory cytokines (Ryan and Kelly, 2016). Together, these results suggest that spending any time in daily lifestyle activity above sedentary behavior that is accrued in a continuous manner may be indicative of higher cognitive performance but the directionality of this relationship requires further evaluation with longitudinal research.

Existing research suggests that self-reported engagement in sedentary behaviors is associated with poorer cognitive performance (Falck et al., 2017). In 2015, Steinberg and colleagues reported that, in a sample of healthy older adults, sedentary pastimes (e.g., computer use) were negatively associated with cognitive performance, primarily in terms of executive function (Steinberg et al., 2015). Additionally, Kesse-Guyot et al. (2012) showed that higher television watching was associated with poorer executive functioning in healthy older adults. However, these studies used self-reported sedentary behaviors; a tool heavily subjected to social desirability and recall biases that are highly susceptible to inaccuracies in people with cognitive deficits (Eshkoor et al., 2015). Our findings with objectively measured sedentary time suggest a relatively minor association with cognitive function – e.g., only one significant association with DSC was found. A possible explanation for the differential findings is that accelerometer data capture all sedentary activities as opposed to a single behavior such as television viewing noted in previous studies. More recent studies that utilize accelerometer data to extract sedentary behaviors support our findings. A recent cross-sectional study in 114 older adults who reside in assisted living showed no association between total sedentary time and global cognition (Leung et al., 2017). Further, another observational study conducted in 2017 in middle-to-older aged US Hispanic/Latinos showed that accelerometer-based sedentary time was nearly associated with DSC but not statistically significance after full covariate adjustment (Vásquez et al., 2017). However, it is important to note study differences by inclusion criteria driven by differences in study purpose and design along with the use of different types of accelerometers may explain why we found a significant relationship between total sedentary time and DSC. More studies are needed to replicate our findings, measure time spent in each type of sedentary behavior, and explore each behavior’s effect on cognitive health.

Intervention results for the LIFE study demonstrated that a 24-month moderate intensity PA program, when compared to a health education program, did not result in improvements in global or domain-specific cognitive function, nor did it alter the incidence of mild cognitive impairment or dementia (Sink et al., 2015). Additionally, the LIFE study had little impact on patterns of sedentary times (Wanigatunga et al., 2017a). Therefore, it is important to convey the potential reasons why the intervention-based findings seemingly contradict the results presented here. First, results from the current study reflect pre-intervention activity and sedentary patterns, which are a product of long-term, or even lifelong activity levels. As shown in previous reports, current activity status is a consistent predictor of past exercise behavior and exercise habits (for a review see Dishman et al., 1985). Second, the PA intervention was compared to a health education group who received social stimulation which may have combated cognitive decline over time. In fact, there was little evidence for cognitive decline in either intervention. Third, the intervention focused on increasing moderate-intensity PA, while the current analysis is focused on accumulation of activity at all intensities and sedentary behaviors. As we showed previously, most activity (>85%) is accumulated at light intensities and our results suggest that this category may be a target for future intervention efforts (Wanigatunga et al., 2017b).

Strengths of the present study include a clinically relevant and large sample of older adults at risk for mobility disability and cognitive decline. Additional strengths include a battery of cognitive assessments, objective movement-based activity data through hip-worn accelerometry, and a multitude of demographics, behavioral, anthropometric, and medical history data. However, a limitation to the current study was the inability to determine a longitudinal relationship between activity/sedentary patterns and cognitive outcomes using a cross-sectional design. Additional research is needed to attempt to elucidate a causal relationship and potential mechanisms that explain how activity/sedentary lifestyle patterns contribute to cognitive impairment and possibly dementia with aging. Another limitation is the generalizability of the results to an older adult population is restricted because the LIFE study excluded those who were either physically well-functioning or severely cognitively impaired. Older adults with low cognition were screened out of the LIFE study to ensure adherence to the study sessions and compliance with the study protocols. Further, LIFE participants were already sedentary and excluded if they reported >20 min per week in moderate-to-vigorous PA. As such, stronger associations between activity and sedentary patterns with cognition may be observed in a population-based sample rather than a sample of older adults with high of disability.

Overall, this research found that higher amounts of PA was positively associated with psychomotor speed, attention, and working memory performance. Individuals who accumulated PA in longer bouts showed better performance on measures of verbal learning and memory. Thus, the impact of PA on memory function is potentially influenced by way its accumulated. Alternatively, it could also be interpreted that those with more preserved cognitive function have the capability to be active for longer continuous periods of time as in accomplishing a task-oriented goal. Another finding from this study was that time spent being sedentary was largely not associated with multiple measures of cognitive function. Future research is needed to better characterize longitudinal changes in the accumulation of daily activity and sedentary behaviors as potential biophysical markers or intervention targets of cognitive status in older adults.

## Data Availability

The raw data supporting the conclusions of this manuscript will be made available by the authors, without undue reservation, to any qualified researcher.

## Author Contributions

AW, TM, ME, and JN contributed to the conceptual design, acquisition, analysis, and interpretation of the data and also the writing of the manuscript. DC, JK, RF, AFK, JV, SR, KS, ACK, TB, SA, NN, JJ, KR, ME, TG, and MP contributed to the interpretation and writing of the manuscript. AW, TM, and JN contributed to revising the work critically and the final approval of the work.

## Conflict of Interest Statement

The authors declare that the research was conducted in the absence of any commercial or financial relationships that could be construed as a potential conflict of interest. Any opinions, findings, conclusion, or recommendations expressed in this publication are those of the author(s) and do not necessarily reflect the view of the United States Department of Agriculture.
